# Assessment of extensor hallucis brevis stiffness and microcirculation in diabetes: shear wave elastography and contrast-enhanced ultrasound

**DOI:** 10.3389/fendo.2025.1639270

**Published:** 2025-07-23

**Authors:** Fuqiang Yan, Mingli Cai, Meirong Li, Shanshan Huang, Jingyi Guo, Jinmiao Lin, Guorong Lyu

**Affiliations:** ^1^ Department of Ultrasound Medicine, Jinjiang Municipal Hospital (Shanghai Sixth People’s Hospital), Quanzhou, China; ^2^ Department of Endocrinology, Jinjiang Municipal Hospital (Shanghai Sixth People’s Hospital), Quanzhou, China; ^3^ Department of Ultrasound Medicine, The Second Affiliated Hospital of Fujian Medical University, Quanzhou, China

**Keywords:** diabetes, skeletal muscle, microcirculation, elasticity, ultrasound diagnosis

## Abstract

**Background:**

Diabetic foot complications, driven by microvascular dysfunction, remain a leading cause of morbidity and amputations. Early detection of microcirculatory and biomechanical alterations in vulnerable muscles, such as the extensor hallucis brevis (EHB), may contribute to risk stratification. However, noninvasive tools for quantifying these changes are lacking.

**Methods:**

This cross-sectional study enrolled 90 participants stratified into healthy controls, uncomplicated type 2 diabetes (T2DM), and T2DM with microvascular complications (MC). Shear wave elastography (SWE) measured EHB stiffness (mean Young’s modulus, Emean), while contrast-enhanced ultrasound (CEUS) assessed perfusion dynamics (transcapillary transit time [ΔAT], net enhancement intensity [ΔPI]). Diagnostic accuracy and reproducibility were evaluated via ROC analysis and intra-class correlation coefficients (ICC).

**Results:**

Emean increased progressively across groups (control: 11.88 kPa; T2DM: 15.78 kPa; T2DM+MC: 18.57 kPa; P < 0.01). T2DM+MC exhibited prolonged ΔAT (89.5 s vs. 50.5 s in controls) and reduced ΔPI (5.0 dB vs. 7.0 dB; P < 0.01). ROC analysis demonstrated high diagnostic accuracy for ΔAT (AUC = 0.970), Emean (AUC = 0.947), and ΔPI (AUC = 0.931) in detecting MC. Both SWE and CEUS showed excellent reproducibility (ICC > 0.80).

**Conclusion:**

SWE and CEUS provide robust, noninvasive biomarkers for early diabetic microvascular complications. The EHB’s unique susceptibility to stiffness and perfusion deficits highlights its clinical value, which may facilitate diabetic foot risk assessment and guide timely interventions to mitigate ulceration and amputations.

## 1 Introduction

Diabetic foot, a debilitating complication of diabetes mellitus, poses significant clinical challenges due to its association with high amputation rates, escalating healthcare costs, and profound deterioration in patient quality of life (1). Its pathogenesis is intricately linked to microvascular dysfunction, which disrupts tissue perfusion and accelerates end-organ damage (2). Skeletal muscle, as a highly vascularized tissue (3), represents a critical yet underexplored target for evaluating diabetes-related microcirculatory impairment. Advances in ultrasound imaging have positioned this modality as a pivotal tool for assessing skeletal muscle architecture and predicting clinical outcomes (4). Notably, contrast-enhanced ultrasound (CEUS) enables dynamic tracking of microvascular perfusion (5, 6), while shear wave elastography (SWE) quantifies tissue stiffness as a surrogate for muscle quality and biomechanical function (7, 8). However, existing studies predominantly focus on larger lower-limb muscles, such as the gastrocnemius, leaving smaller dorsal foot muscles—particularly the extensor hallucis brevis (EHB)—poorly characterized in the context of diabetes.

The EHB, originating from the calcaneus and inserting into the proximal phalanx of the hallux (9), exhibits distinct anatomical and hemodynamic features compared to proximal muscles: reduced volumetric perfusion, smaller cross-sectional area, and susceptibility to early microvascular compromise (9). These attributes render the EHB an ideal candidate for studying distal microcirculatory pathology in diabetes. Despite the EHB’s clinical relevance to diabetic foot complications, standardized imaging protocols and diagnostic biomarkers for early microvascular compromise in this muscle remain scarce. CEUS and SWE offer rapid, noninvasive, and cost-effective alternatives for simultaneous assessment of microvascular perfusion and tissue stiffness—capabilities critical for early detection of diabetic myopathy.

This study aims to address this gap by leveraging CEUS and SWE to characterize microcirculatory dynamics and biomechanical properties of the EHB across diabetic stages. We hypothesize that progressive microvascular dysfunction and fibrotic remodeling in diabetes will manifest as quantifiable alterations in CEUS-derived perfusion parameters and SWE-measured tissue stiffness. By establishing objective imaging biomarkers, this work seeks to enhance early diagnosis and risk stratification for diabetic foot complications, ultimately informing targeted therapeutic strategies.

## 2 Materials and methods

### 2.1 Study participants

This prospective cross-sectional study recruited 90 participants from Jinjiang Municipal Hospital (affiliated as the Fujian Campus of Shanghai Sixth People’s Hospital) between June 2020 and March 2023. Participants were stratified into three groups:

T2DM without complications (DM group): Thirty patients diagnosed with uncomplicated type 2 diabetes mellitus, including 22 individuals with comorbid hypertension and 18 with dyslipidemia.
T2DM with microvascular complications (DM+MC group): Thirty patients with type 2 diabetes and confirmed microvascular complications, defined as diabetic nephropathy (n = 15), diabetic retinopathy (n = 12), and/or peripheral neuropathy (n = 24). Within this group, 23 participants had hypertension and 17 exhibited dyslipidemia.
Control group: Thirty age- and sex-matched healthy volunteers with no history of diabetes or metabolic disorders.

The demographic characteristics (age, sex distribution) of each group are summarized in **Table 1**. Diagnoses of type 2 diabetes mellitus (T2DM) and microvascular complications (MC) adhered to the 2019 American Diabetes Association (ADA) Standards of Medical Care. Exclusion criteria included: (1) history of cardiovascular events (e.g., myocardial infarction, heart failure, structural or cardiomyopathic heart disease, severe arrhythmias, stroke) or prior cardiocerebrovascular surgical interventions; (2) diagnosed peripheral vascular disease; (3) active autoimmune disorders; (4) current or history of malignancies; (5) pregnancy; (6) suboptimal imaging quality or participant noncompliance during examinations.

**Table 1:** Comparison of clinical characteristics among study groups.

Characteristics	Normal group (n = 30)	DM group (n = 30)	DM+MC group (n = 30)	*P*-value
Sex (male/female), n	14/16	15/15	14/16	0.96
Age (years)	53.8 ± 8.9	55.8 ± 10.4	57.9 ± 11.3	0.31
BMI (kg/m²)	22.3 ± 3.0	23.6 ± 3.0	23.3 ± 2.4	0.21
Disease duration (years)^a^	0	6.0 (1.0-10.0)	10.0 (4.8-12.0)^*^	<0.01
Fasting glucose (mmol/L)^a^	4.7 (3.9-5.1)	9.1 (7.8-12.4)	11.5 (10.1-16.3)^*^	<0.01
HbA1c (%)^a^	5.0 (4.1-5.5)	7.7 (6.9-10.9)	11.9 (9.4-13.6)^*^	<0.01

Data presented as *mean ± SD* or ^a^ *median (IQR)*.

DM, type 2 diabetes; DM+MC, DM with microvascular complications; BMI, body mass index; HbA1c, glycated hemoglobin.

*Post-hoc* comparisons: ^*^ *P* < 0.05 vs. DM group.

Muscle imaging data were systematically acquired from the right EHB to ensure anatomical consistency across assessments.

### 2.2 Methods

#### 2.2.1 Clinical data collection

Demographic and clinical parameters, including sex, age, body mass index (BMI), blood pressure, diabetes duration, and complications, were systematically recorded. Laboratory assessments encompassed fasting plasma glucose (FPG), and glycated hemoglobin (HbA1c).

#### 2.2.2 Ultrasound equipment

Musculoskeletal ultrasound examinations were conducted using a Mindray Resona R9 system (Mindray, Shenzhen, China) equipped with an L-14 linear array transducer (5.0–12.0 MHz). Imaging parameters were standardized across all participants: mechanical index (MI) = 1.4, gain = 60%, imaging depth = 2.5 cm, and single focal zone positioned at 0.6 cm. For CEUS, sulfur hexafluoride microbubble contrast agent (SonoVue^®^; Bracco Imaging, Milan, Italy) was reconstituted with 5.0 mL of sterile saline solution according to the manufacturer’s instructions and vigorously shaken to form a homogeneous microbubble suspension. A 2.5 mL bolus of the prepared SonoVue^®^ suspension was then administered intravenously via the antecubital vein, followed by a 5.0 mL saline flush.

#### 2.2.3 Shear wave elastography

Participants were positioned supine with knees flexed and feet flat on the examination table to ensure passive relaxation of the toe extensor muscles. After applying a water-based coupling gel to the right EHB, the transducer was gently placed over the muscle belly without exerting pressure to minimize compression artifacts. The EHB was identified in its long-axis view, with the superior border anchored at the calcaneal attachment site. SWE mode was activated following probe stabilization, and measurements were initiated only when the system’s stability index reached 5 stars. A 3-mm circular region of interest (ROI) was positioned at the mid-portion of the EHB to quantify the Emean (kPa). Three consecutive measurements were obtained and averaged for final analysis. In the control group, SWE assessments were independently performed by two senior sonographers to evaluate inter-observer reproducibility.

#### 2.2.4 Contrast-enhanced ultrasound

Participant positioning and transducer placement mirrored SWE protocols. A longitudinal view of the EHB was acquired, and the probe was rotated 90° at 1–2 cm distal to the calcaneal attachment to visualize the lateral tarsal artery. A 2.5 mL bolus of SonoVue^®^ was injected intravenously via the antecubital vein, followed by a 5.0 mL saline flush. Dynamic CEUS cine loops were recorded continuously for 180 seconds.

Time-intensity curves (TICs) were generated offline using dedicated software (Mindray QLAB, version 10.8). Two ROIs were analyzed: (1) within the EHB parenchyma, avoiding fascial boundaries or non-muscular regions, and (2) surrounding the lateral tarsal artery. The following parameters were extracted: arrival time (AT, s; time from contrast injection to first detectable signal in the EHB), transcapillary transit time (ΔAT, s; time difference between arterial and muscular contrast arrival), baseline intensity (BI, dB), peak intensity (PI, dB), and net enhancement intensity (ΔPI, dB; calculated as PI − BI). Triplicate measurements were averaged for statistical analysis. In the control group, CEUS data were independently analyzed by two blinded sonographers to assess inter-observer reliability.

### 2.3 Statistical analysis

Data were analyzed using SPSS 26.0 (IBM Corp.). Continuous variables underwent normality testing via the Kolmogorov-Smirnov test and homogeneity of variance via Levene’s test. Normally distributed data are expressed as mean ± SD. Multi-group comparisons employed one-way ANOVA (for homogeneous variance) or Welch ANOVA (heterogeneous variance), followed by *post hoc* LSD-t test (homogeneous variance) or Tamhane’s T2 test (heterogeneous variance). Non-normally distributed data are reported as median (IQR) and analyzed with the Kruskal-Wallis H test, with Bonferroni correction for pairwise comparisons. Categorical variables are presented as counts.

Receiver operating characteristic (ROC) curves were constructed to evaluate the diagnostic efficacy of SWE and CEUS parameters for diabetic microvascular complications. DeLong’s test compared the areas under the ROC curves (AUCs). Intra- and inter-observer reproducibility of SWE and CEUS measurements was assessed using intra-class correlation coefficients (ICC), with ICC > 0.75 indicating good reproducibility and ICC < 0.40 indicating poor reproducibility. A two-tailed P < 0.05 defined statistical significance.

## 3 Results

### 3.1 Comparison of baseline characteristics among groups

As shown in **Table 1**. No significant differences in sex, age, or BMI were observed across the control, DM, and DM+MC groups (*P* > 0.05). However, diabetes duration, fasting plasma glucose (FPG), and glycated hemoglobin (HbA1c) differed significantly among the three groups (*P* < 0.05). The DM+MC group exhibited higher values than the DM group, and the DM group exceeded the control group, with all pairwise comparisons reaching statistical significance (*P* < 0.05).

### 3.2 Comparison of SWE and CEUS parameters among groups

**Figure 1:**
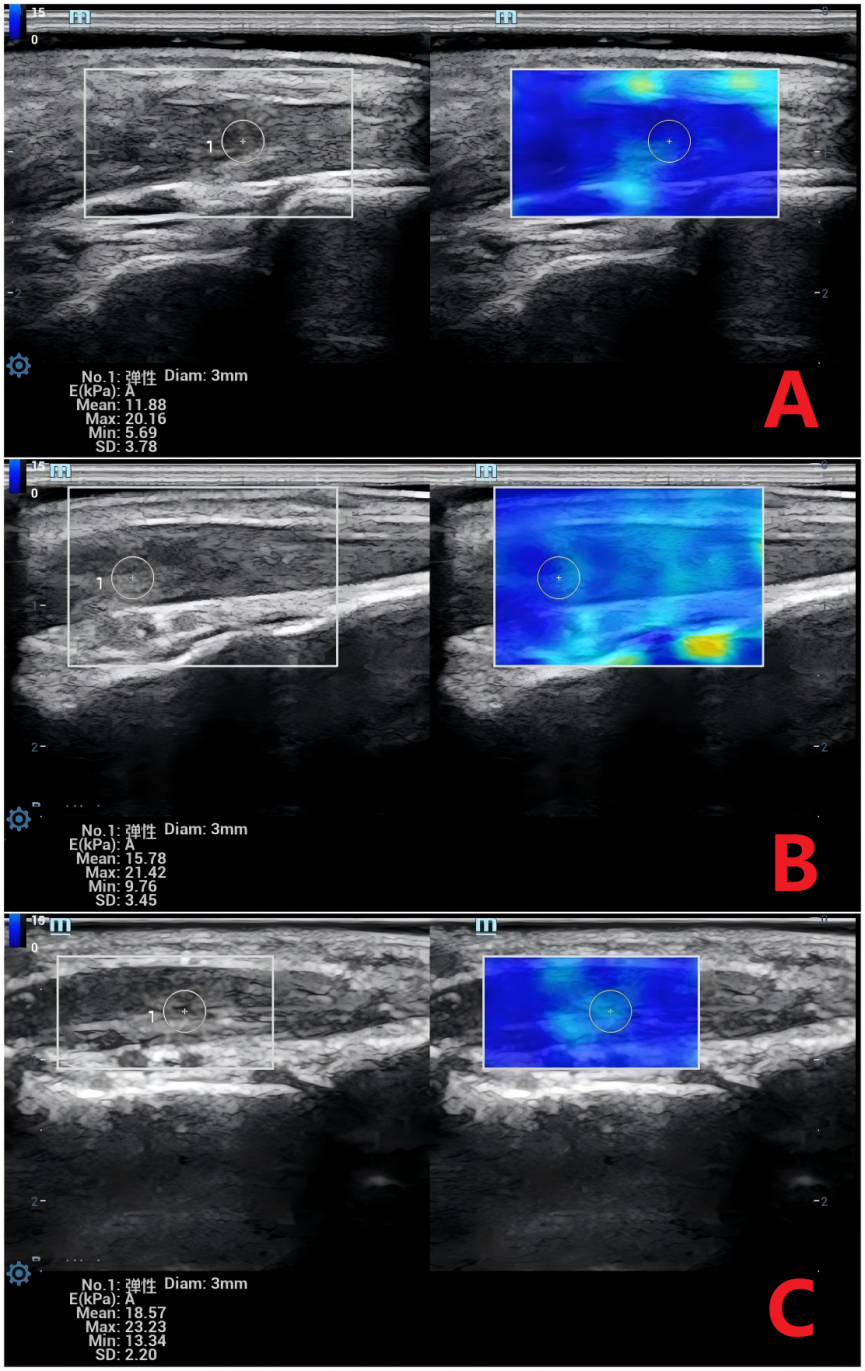
Shear Wave Elastography (SWE) Images of the Extensor Hallucis Brevis Muscle Across Groups. **(A)** Normal group: Mean Young’s modulus (Emean) = 11.88 kPa. **(B)** DM group: Emean = 15.78 kPa. **(C)** DM+MC group: Emean = 18.57 kPa. DM, Type 2 diabetes; DM+MC, DM with microvascular complications. SWE measurements reflect increasing muscle stiffness with disease progression.

**Figure 2:**
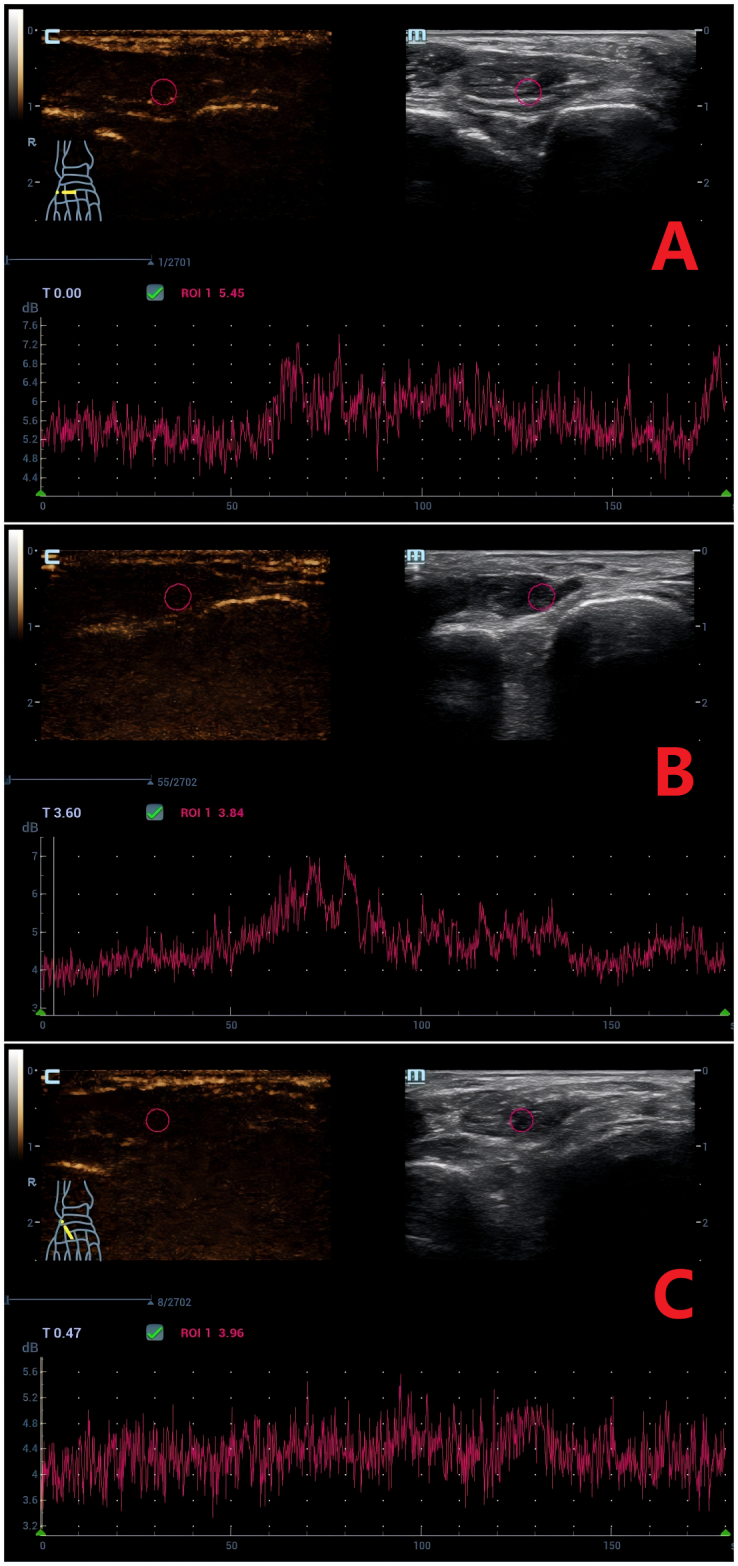
Time-Intensity Curves (TICs) of Contrast-Enhanced Ultrasound (CEUS) for the Extensor Hallucis Brevis Muscle. **(A)** Normal group: Arrival time (AT) = 50.5 s, peak intensity (PI) = 7.0 db. **(B)** DM group: AT = 51.6 s, PI = 6.7 db. **(C)** DM+MC group: AT = 89.5 s, PI = 5.0 db. CEUS parameters demonstrate delayed contrast arrival (AT) and reduced perfusion (PI) in the DM+MC group.

SWE and CEUS parameters are summarized in **Table 2**. BI showed no intergroup differences (*P* > 0.05). Significant variations were detected in Emean, PI, ΔPI, AT, and ΔAT (*P* < 0.05). Pairwise analyses revealed: Emean was higher in the DM+MC group than in the DM and control groups, and higher in the DM group than in controls (*P* < 0.05). PI and ΔPI were lower in the DM+MC group compared to the DM and control groups, with the DM group also lower than controls (*P* < 0.05). AT and ΔAT were prolonged in the DM+MC group versus both the DM and control groups (*P* < 0.05), but no differences were observed between the DM and control groups (*P* > 0.05). Representative images demonstrating the progressive increase in Emean across diabetic stages are shown in **Figure 1**, while perfusion characteristics are visualized in **Figure 2**. 

**Table 2:** Comparison of SWE and CEUS parameters among groups.

Parameter	Normal group (n=30)	DM group (n=30)	DM+MC group (n=30)	Statistic (F/H)	*P*-value
Emean (kPa)	12.35 (11.35-13.03)	14.00 (13.02-15.10)^a^	17.50 (15.30-19.30)^ab^	*H* = 60.13	<0.01
BI (dB)	3.70 (3.30-4.00)	3.70 (3.30-4.00)	3.75 (3.35-3.93)	*H* = 0.039	0.98
PI (dB)	7.27 ± 0.63	6.65 ± 0.41^a^	5.77 ± 0.86^ab^	*F* = 39.63	<0.01
ΔPI (dB)	3.64 ± 0.55	3.00 ± 0.40^a^	2.18 ± 0.50^ab^	*F* = 67.65	<0.01
AT (s)	50.35 (42.95-55.00)	49.85 (44.62-55.65)	57.20 (50.67-67.00)^ab^	*H* = 12.42	<0.01
ΔAT (s)	10.20 (9.75-11.30)	12.30 (11.15-13.92)	21.35 (18.02-28.07)^ab^	*H* = 57.60	<0.01

Data presented as median (IQR) or mean ± SD.

SWE, Shear wave elastography; CEUS, Contrast-enhanced ultrasound; Emean, Mean Young’s modulus; BI, Baseline intensity; PI, Peak intensity; ΔPI, Net enhancement intensity (PI − BI); AT, Arrival time; ΔAT, Transcapillary transit time.

a Significantly different vs. Normal group (P < 0.05); Significantly different vs. DM group (P < 0.05).

### 3.3 Diagnostic performance of SWE and CEUS for diabetic microvascular complications

**Figure 3:**
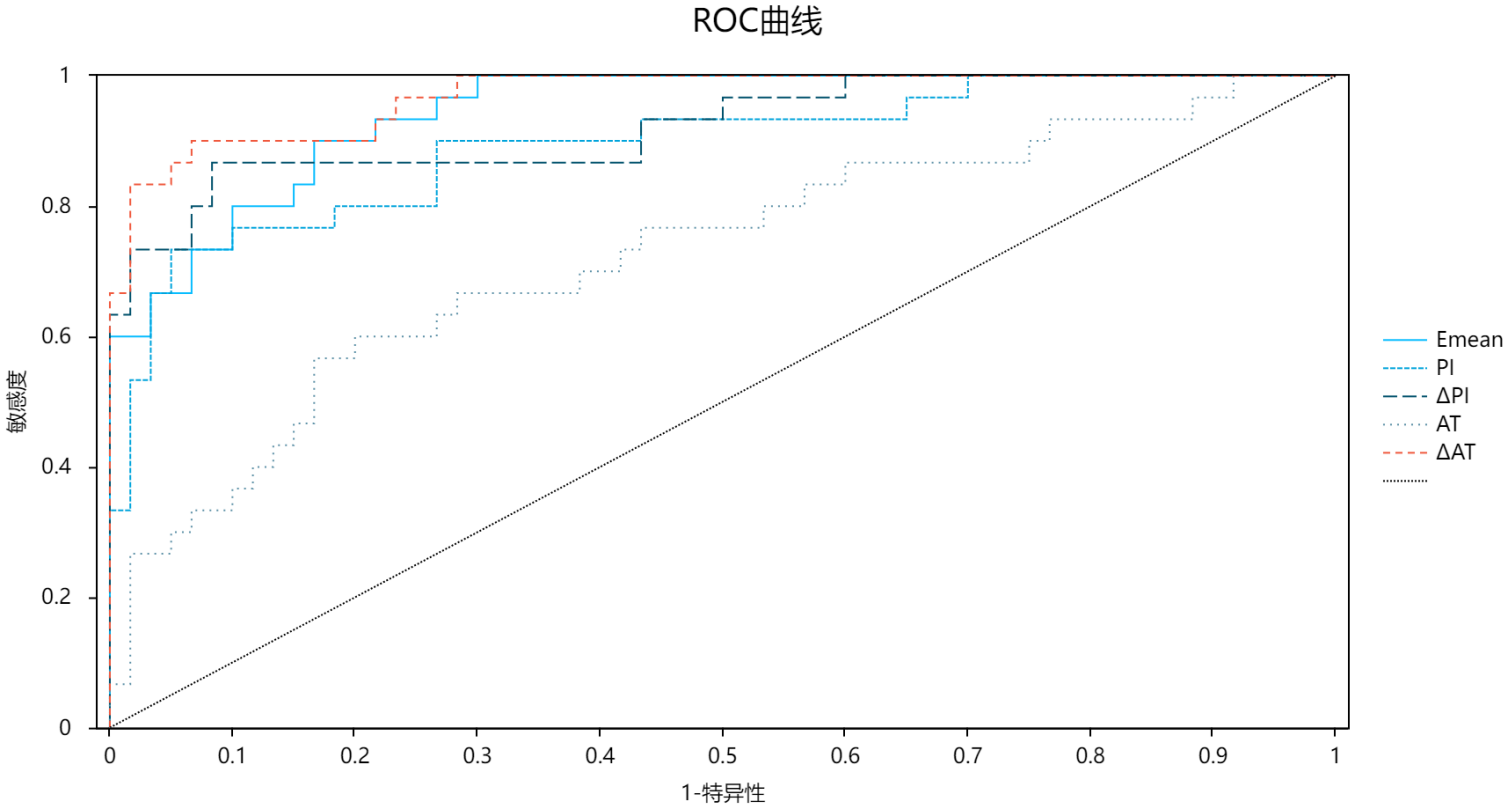
ROC curve chart comparing five diagnostic parameters: Emean, PI, API, AT, and AAT. Sensitivity is on the y-axis, and 1-specificity on the x-axis. A diagonal reference line indicates random chance. Curves demonstrate high diagnostic accuracy for AAT (AUC=0.970), Emean (AUC=0.947), and API (AUC=0.931) in detecting diabetic microvascular complications.

ROC analysis (**Table 3**; **Figure 3**) demonstrated high diagnostic efficacy for ΔAT (AUC = 0.970), Emean (AUC = 0.947), and ΔPI (AUC = 0.931), with no significant AUC differences among these parameters (*P* > 0.05). While Emean and ΔPI showed numerically higher AUCs than PI, the differences were nonsignificant (*P* > 0.05). All three parameters significantly outperformed AT (*P* < 0.05). Optimal cutoffs and diagnostic metrics were: ΔAT: 14.80 (sensitivity 90.0%, specificity 93.3%). Emean: 14.6 kPa (sensitivity 90.0%, specificity 83.3%). ΔPI: 2.70 dB (sensitivity 86.7%, specificity 91.7%).

**Table 3:** Diagnostic performance of SWE and CEUS parameters for detecting diabetic microvascular complications.

Parameter	AUC (95% CI)	Cut-off	Sensitivity (%)	Specificity (%)	*P*-value^vs. AT^
ΔAT	0.970 (0.925–1.000)	14.80 s	90.0	93.3	<0.05
Emean	0.947 (0.890–0.995)	14.60 kPa	90.0	83.3	<0.05
ΔPI	0.931 (0.868–0.994)	2.70 dB	86.7	91.7	<0.05
PI	0.904 (0.834–0.974)	6.20 dB	73.3	95.0	<0.05
AT	0.729 (0.612–0.846)	56.70 s	56.7	83.3	–

AUC, Area under the ROC curve; ΔAT, Transcapillary transit time; Emean, Mean Young’s modulus; ΔPI, Net enhancement intensity; AT, Arrival time.

Statistical comparisons by DeLong test: P-values indicate difference vs. AT (reference parameter).

### 3.4 Reproducibility of SWE and CEUS measurements

As shown in **Table 4**, both inter- and intra-observer reproducibility for SWE measurements were excellent (ICC > 0.80), with intra-observer consistency slightly superior to inter-observer agreement.

**Table 4:** Intra- and inter-observer reliability of SWE and CEUS measurements.

Parameter	Intra-observer ICC (95% CI)	Inter-observer ICC (95% CI)
Emean	0.861 (0.708–0.934)	0.803 (0.585–0.906)
BI	0.860 (0.706–0.934)	0.808 (0.597–0.909)
PI	0.972 (0.941–0.987)	0.943 (0.881–0.973)
ΔPI	0.967 (0.930–0.984)	0.761 (0.498–0.886)
AT	0.991 (0.981–0.996)	0.985 (0.968–0.993)
ΔAT	0.970 (0.936–0.986)	0.966 (0.928–0.984)

ICC, Intraclass correlation coefficient; SWE, Shear wave elastography; CEUS, Contrast-enhanced ultrasound; Emean, Mean Young’s modulus (kPa); BI, Baseline intensity (dB); PI, Peak intensity (dB); ΔPI, Net enhancement intensity (dB); AT, Arrival time (s); ΔAT, Transcapillary transit time (s).

Interpretation: ICC > 0.75 = good reliability; ICC > 0.90 = excellent reliability.

## 4 Discussion

Globally, approximately 18.6 million individuals develop diabetic foot ulcers annually. Notably, 80% of lower extremity amputations in diabetic patients are attributable to these ulcers, which are strongly associated with an elevated mortality risk (10). The dorsum of the foot is a high-risk site for ulceration (11), a vulnerability driven by the progressive nature of diabetic microvascular complications. Specifically, early-stage disease predominantly affects distal small arteries before advancing to medium- and large-caliber vessels (12). Consequently, early detection of dorsum-specific microvascular dysfunction is critical for mitigating severe outcomes in diabetes.

Current diagnostic modalities for dorsal foot evaluation include clinical examination combined with laser Doppler flowmetry, capillary microscopy, and optical coherence tomography—methods that reliably assess cutaneous elasticity and microcirculation (13). For deeper musculature, radiotracer imaging and MRI are effective in detecting microvascular perfusion abnormalities in diabetic skeletal muscle (14, 15), with MRI additionally quantifying tissue stiffness (16). However, the prohibitive costs and prolonged acquisition times of these imaging techniques limit their routine clinical utility. In contrast, ultrasound-based techniques, particularly CEUS and SWE, offer a safe, rapid, and cost-effective alternative. These well-validated modalities enable simultaneous assessment of microvascular perfusion and tissue stiffness, making them scalable tools for widespread clinical adoption.

Our findings demonstrate significant reductions in PI and ΔPI, alongside prolonged AT and ΔAT in the DM+MC group compared to controls. These observations likely reflect the underlying pathophysiology of progressive diabetes. Specifically, advanced microvascular complications are characterized by severe endothelial dysfunction and microvascular occlusion due to accumulated advanced glycation end products (AGEs), which impede microbubble transit, thereby reducing perfusion efficiency. This aligns with the findings of Zheng et al. (17), who observed diminished skeletal muscle microcirculation and delayed contrast arrival in diabetic rabbit models. Histopathological analysis in their study further revealed disrupted myofilament architecture, myolysis, and mitochondrial degeneration, with severity correlating with disease duration.

Notably, while the DM group exhibited reduced PI and ΔPI relative to controls, AT and ΔAT remained comparable. We hypothesize that early-stage diabetic microangiopathy involves vascular remodeling without critical stenosis. This permits unimpeded contrast arrival but reduces total perfused volume due to mild luminal narrowing. Such a mechanism is corroborated by Jefferson C. Frisbee et al. (18) who documented microvascular rarefaction in prediabetic rodent skeletal muscle without concurrent structural or functional compromise. Beyond diagnostic applications, recent advances in nanoformulations—such as insulin-loaded nanoparticles optimizing glycemic control (19), antioxidant-loaded nanocomposites mitigating oxidative stress (20), and glucose-responsive nanocarriers enhancing tissue perfusion (21)—demonstrate synergistic potential with early microcirculatory assessment. While our study focuses on diagnostic biomarkers, these multidisciplinary innovations highlight future opportunities for combined diagnostic-therapeutic strategies targeting microvascular pathology.

Our study revealed a progressive increase in Emean measured by SWE, with the highest values observed in the DM+MC group. This suggests that diabetic severity correlates with elevated stiffness of the EHB, likely attributable to skeletal muscle atrophy and fibrotic remodeling (22). Ischemic mechanisms may contribute, as diabetic microvascular dysfunction can impair skeletal muscle perfusion, culminating in ischemic atrophy in advanced stages (23). Metabolically, insulin resistance in type 2 diabetes reduces protein synthesis, exacerbating muscle wasting (24). Furthermore, activation of PDGFRα signaling and glycolytic pathways by CD34+CD90+ fibro-adipogenic progenitors (FAPs) in diabetic individuals may drive fibrotic infiltration within muscle tissue (25).

Notably, prior studies on larger muscles (e.g., gastrocnemius) in diabetic populations reported reduced stiffness with disease progression, regardless of sarcopenia status (26, 27), contrasting with our EHB findings. We hypothesize that the EHB’s distal location, smaller volume, and diminished vascular supply compared to proximal muscles (9) render it more vulnerable to microcirculatory impairment. This vulnerability may accelerate atrophy and fibrosis over compensatory glycolytic adaptation, thereby increasing stiffness. Alternatively, the observed discrepancy could reflect limited sample size or anatomical heterogeneity.

ROC curve analysis demonstrated robust diagnostic performance of ΔAT, Emean, and ΔPI for diabetic microvascular complications. In this study, ΔAT—representing the transcapillary transit time from the lateral tarsal artery to the EHB—provides a more direct measure of regional microcirculatory efficiency than AT (which incorporates systemic venous-to-arterial variability), likely explaining its superior diagnostic accuracy. This aligns with Xu et al. (28), who reported prolonged contrast transit times from artery to gastrocnemius muscle in DM+MC patients compared to DM and control groups. ΔPI, calculated as the net peak intensity (PI − BI), exhibited marginally higher diagnostic efficacy than PI alone, though statistically nonsignificant. This may reflect minimal inter-subject variability in BI under standardized protocols. Notably, Amarteifio et al. (29) similarly observed that ΔPI effectively tracked progressive impairment of skeletal muscle perfusion with advancing diabetes. While the EHB’s stiffness trajectory (Emean increase) contrasts with reported declines in larger muscles, its diagnostic utility remains compelling. We posit that anatomical and hemodynamic distinctions between the EHB and proximal muscles underlie this divergence. Future studies with expanded cohorts will clarify these mechanisms.

SWE measurements are influenced by multiple factors (30). In this study, age, sex, and obesity were matched across groups, and potential confounders—including participant positioning, muscle contraction status, and operator technique—were minimized through standardized protocols: the right EHB was imaged in a relaxed state with uniform probe handling. Reproducibility testing confirmed high intra- and inter-observer agreement (ICC > 0.80), with intra-observer consistency slightly exceeding inter-observer reliability, consistent with Kempfert et al. (31). These results validate the stability of SWE measurements under controlled conditions.

Similarly, CEUS video analyses demonstrated excellent reproducibility, with intra- and inter-observer ICCs > 0.80, aligning with prior studies (6). Collectively, these findings underscore SWE and CEUS as robust, operator-independent tools for quantifying skeletal muscle elasticity and microcirculatory perfusion, supporting their broader clinical implementation.

This study has several limitations. First, its single-center design and relatively small sample size may introduce sampling bias. Multi-center studies with larger, more diverse cohorts are needed to enhance generalizability. Second, muscle biomechanical properties were assessed only under passive conditions, precluding evaluation of dynamic functional correlations during active contraction. Third, patients with diabetic foot ulcers were excluded, limiting insights into end-stage microvascular pathology.

Fourth, the unilateral assessment of the extensor hallucis brevis (EHB) provides localized insights into skeletal muscle microcirculation but does not comprehensively reflect systemic or contralateral muscle status. Furthermore, cutaneous microcirculation—a critical component of diabetic foot pathophysiology—was not addressed. Fifth, while microvascular complications were stratified, neuropathic factors (e.g., peripheral neuropathy) were not rigorously controlled. Future studies should expand sample sizes to disentangle the independent effects of neuropathy on microcirculatory and biomechanical alterations.

## 5 Conclusion

CEUS and SWE effectively characterize microvascular perfusion and biomechanical alterations in the extensor hallucis brevis of patients across the diabetic spectrum. These techniques may enhance clinical risk stratification for diabetic foot complications. We propose targeted EHB screening in long-standing diabetic patients, where Emean >14.6 kPa or ΔAT >14.8 s could prompt early podiatry referrals to mitigate end-stage sequelae. Future multi-center studies with expanded cohorts will validate these thresholds and explore their integration into standardized care pathways.

## Data Availability

The raw data supporting the conclusions of this article will be made available by the authors, without undue reservation.
